# Not Your Usual Suspect: Clear Cell Renal Cell Carcinoma Presenting as Ulcerative Esophagitis

**DOI:** 10.7759/cureus.2821

**Published:** 2018-06-18

**Authors:** Saeed Ali, Basher Atiquzzaman, Konrad Krall, Ranjeet Kumar, Bo Liu, Shantel Hebert-Magee

**Affiliations:** 1 Internal Medicine Residency, Florida Hospital, Orlando, USA; 2 Gastroenterology, Florida Hospital Celebration Health, Celebration, USA; 3 Center for Interventional Endoscopy, Florida Hospital, Orlando, USA; 4 Diagnostic Radiology, Florida Hospital, Orlando, USA; 5 Pathology, Florida Hospital, Orlando , USA

**Keywords:** renal cell carcinoma, metastasis, sunitinib

## Abstract

Renal cell carcinoma (RCC) has the propensity to hematogenously metastasize to the lung, bone, or liver, however, metastasis to the esophagus is exceedingly rare. We report a case of ulcerative esophagitis secondary to recurrent metastatic renal cell cancer status post remote nephrectomy.

An 82-year-old Caucasian male presented with dark tarry stools for two days, progressive dysphagia to solid food for several weeks and unintentional weight loss. His past medical history was significant for hypertension, diverticulosis and right-sided renal cell cancer for which he underwent nephrectomy 13 years ago. Physical examination was unremarkable. Laboratory data showed hemoglobin of 12.5 g/dL, with normal platelet count and an international normalized ratio (INR). His stools were positive for occult blood. Esophagogastroduodenoscopy (EGD) revealed a fragile mid esophageal mass and antral erosive gastritis which were both biopsied. Colonoscopy showed diverticulosis without stigmata of active gastrointestinal (GI) bleed. CT scan (computed tomography) of the chest showed a solid esophageal mass in the lower esophagus as well as a right upper lobe lung mass for which CT-guided needle biopsy was obtained. The histopathology revealed metastatic renal cell cancer of clear cell subtype. The patient was started on palliative radiotherapy. On completion of radiotherapy two months later, his dysphagia had resolved. The patient is currently on chemotherapy with Sunitinib.

Metastatic involvement of esophagus is relatively uncommon and is reported in 6% of patients with metastatic lung, breast and prostate cancer. Esophageal metastasis of clear cell RCC is very rare and so far only seven cases have been reported.

Diagnosis is confirmed by endoscopy, imaging and histopathology. Treatment options include surgical or endoscopic resection for a solitary metastatic lesion. If the tumor is unresectable, multidisciplinary treatment including radiation and chemotherapy is indicated.

## Introduction

The most common types of esophageal malignancies are primary tumors of the esophagus that includes squamous cell carcinoma and adenocarcinoma. Metastasis from other primary tumors is however uncommon. It most commonly occurs from the tumors of adjacent viscera rather than distant tumors. Renal cell carcinoma (RCC) has various subtypes with clear cell carcinoma being the most common subtype. RCC has a tendency to hematogenously metastasize to the lung, bone, or liver, however, metastasis to the esophagus is very rare [1]. Isolated metastasis from RCC has been reported in 2-34% cases after nephrectomy and their surgical resection had shown improvement in overall and long-term survival [2-5].

We report a case of ulcerative esophagitis secondary to metastatic RCC manifesting 13 years after nephrectomy.

## Case presentation

**Figure 1:**
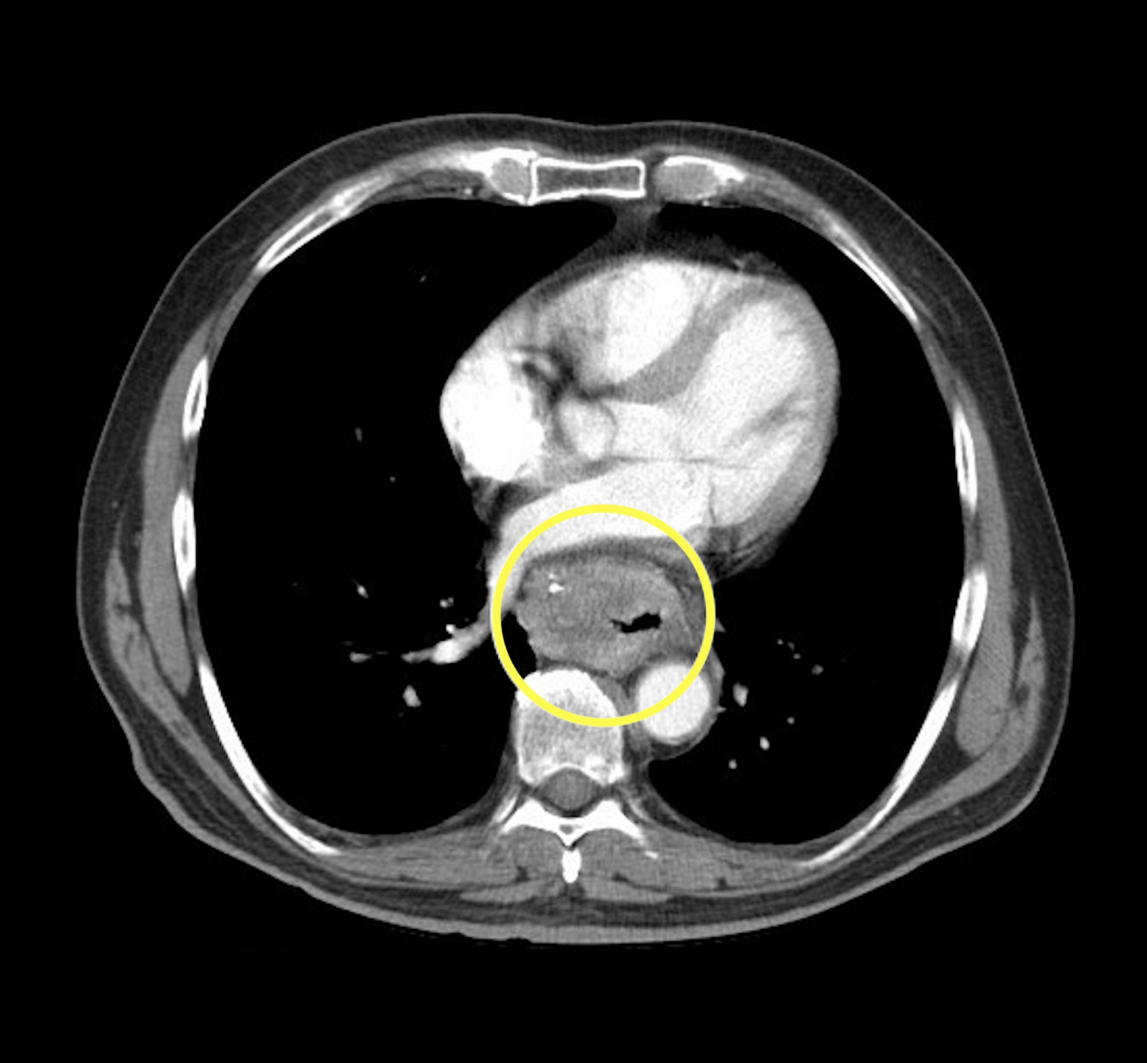
Axial contrast enhanced computed tomography (CT) chest (soft tissue window) demonstrating a solid heterogeneously enhancing esophageal mass (yellow circle).

**Figure 2:**
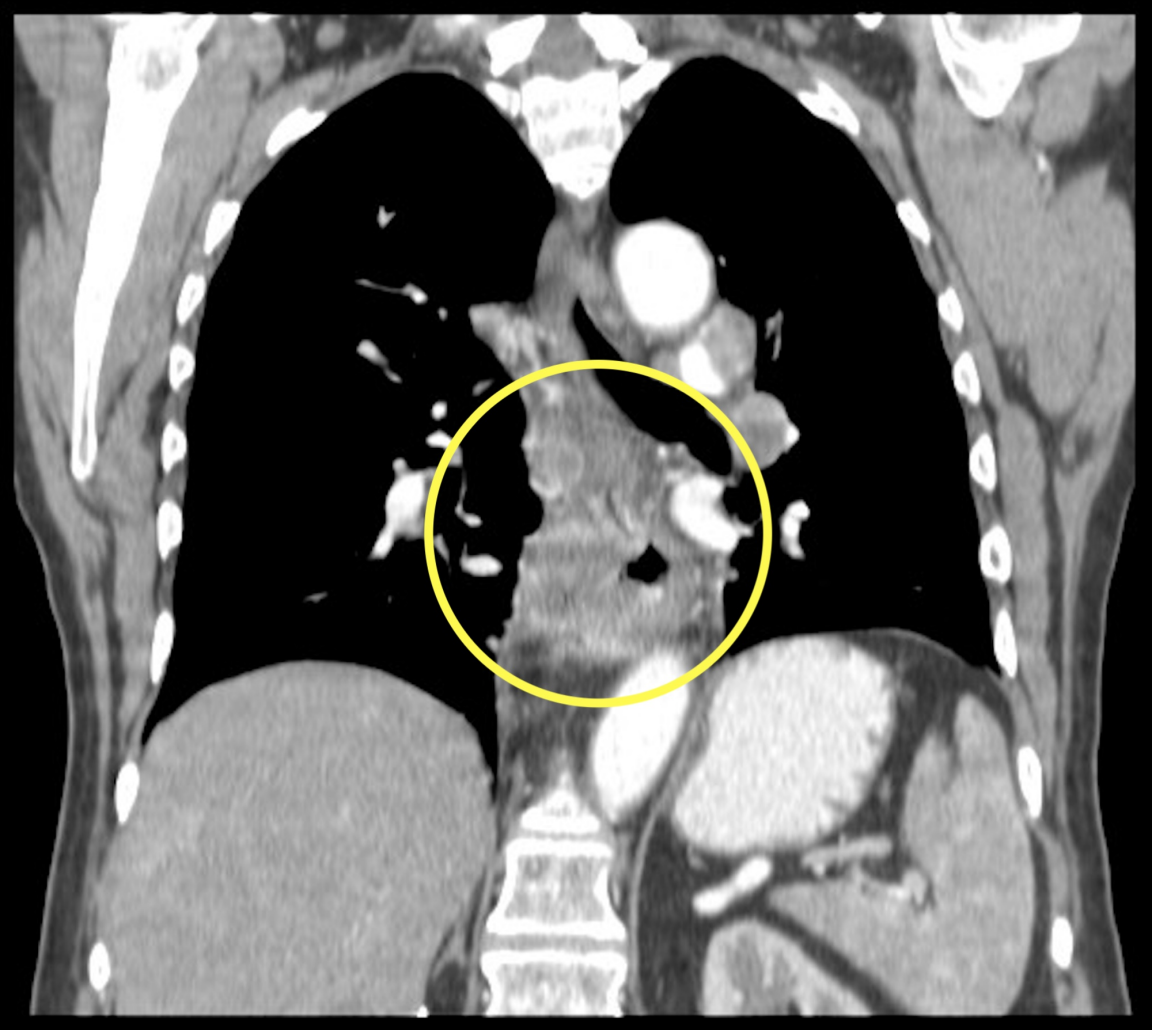
Coronal contrast enhanced computed tomography (CT) chest (soft tissue window) demonstrating a solid heterogeneously enhancing esophageal mass (yellow circle).

**Figure 3:**
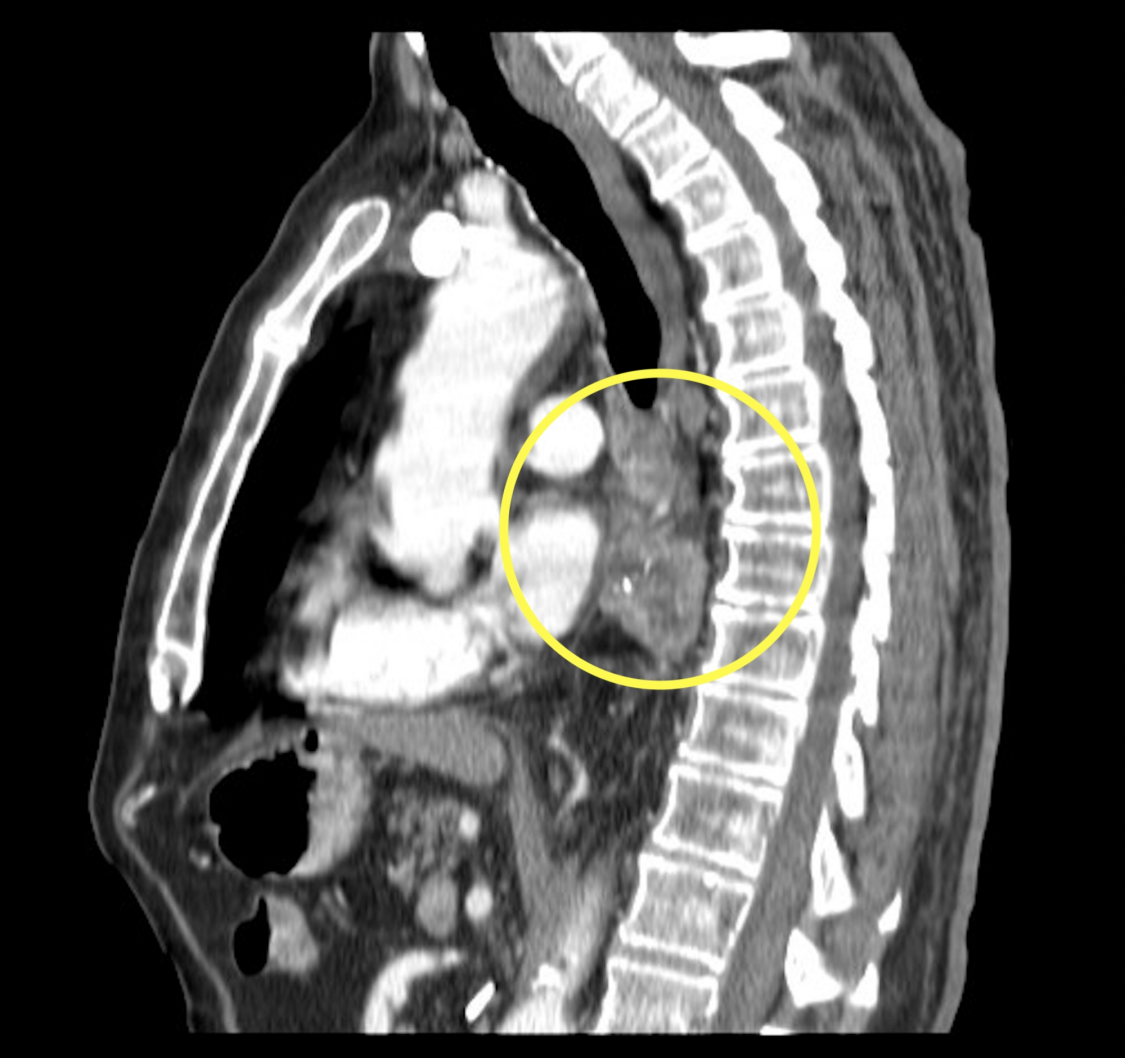
Sagittal contrast enhanced computed tomography (CT) chest (soft tissue window) demonstrating a solid heterogeneously enhancing esophageal mass (yellow circle).

An 82-year-old Caucasian male presented with dark tarry stools for two days, progressive dysphagia to solid food for several weeks and significant unintentional weight loss. His past medical history was significant for hypertension, diverticulosis and right-sided renal cell cancer for which he underwent nephrectomy 13 years ago. He denied family history of gastrointestinal (GI) malignancies. He also denied use of non-steroidal anti-inflammatory drugs, antiplatelet or anticoagulants, smoking, and drinking. Physical examination was unremarkable for hepatosplenomegaly, lymphadenopathy, and blood in the rectal vault. Laboratory data showed a hemoglobin of 12.5 g/dL, with normal platelet count and international normalized ratio (INR). His stools were positive for occult blood. Esophagogastroduodenoscopy (EGD) revealed a fragile mid esophageal mass and antral erosive gastritis which were both biopsied. Colonoscopy showed diverticulosis without stigmata of active GI bleed. Computed tomography (CT) scan of the chest showed a solid esophageal mass in lower esophagus measuring 5 x 4 x 7 cm^3^ (Figures 1-3).

**Figure 4:**
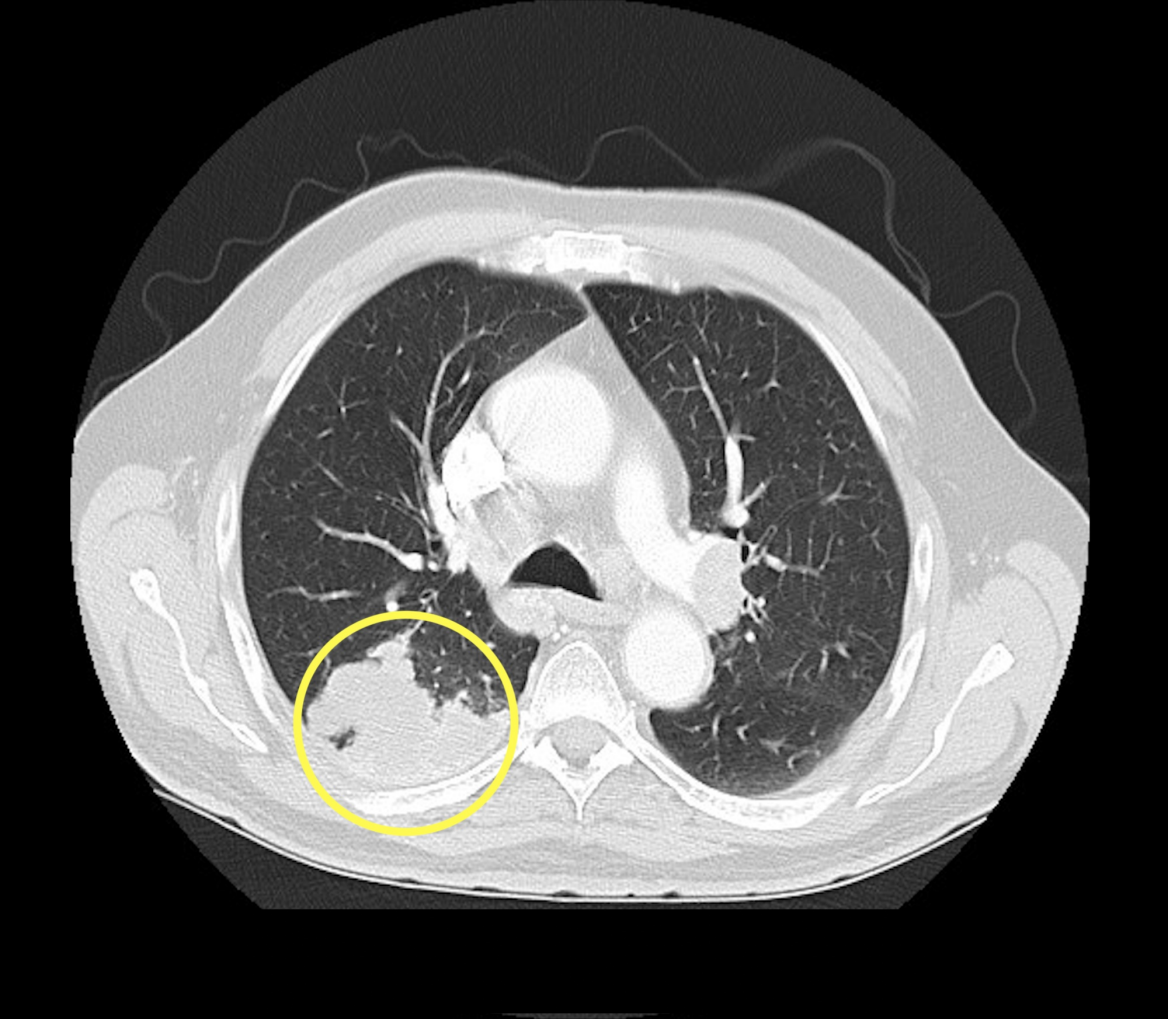
Axial contrast enhanced computed tomography (CT) chest (lung window) demonstrating a lobulated pleural-based mass in the poster segment of the right upper lobe (yellow circle).

It also revealed a right upper lobe lung mass (Figure 4) for which the patient underwent a CT-guided needle biopsy procedure.

**Figure 5:**
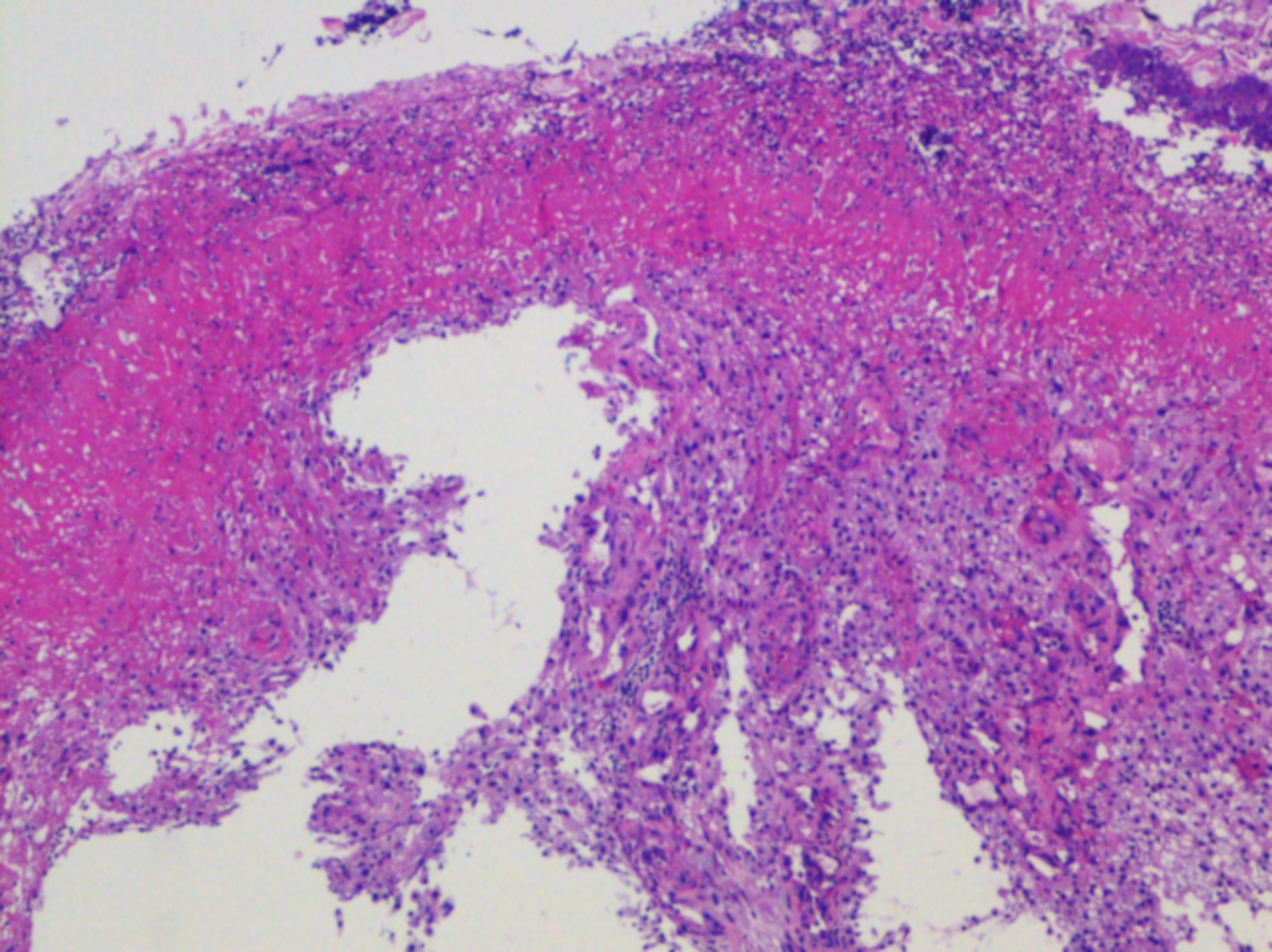
Histopathology from esophageal biopsy showing metastatic clear cell renal cell carcinoma.

**Figure 6:**
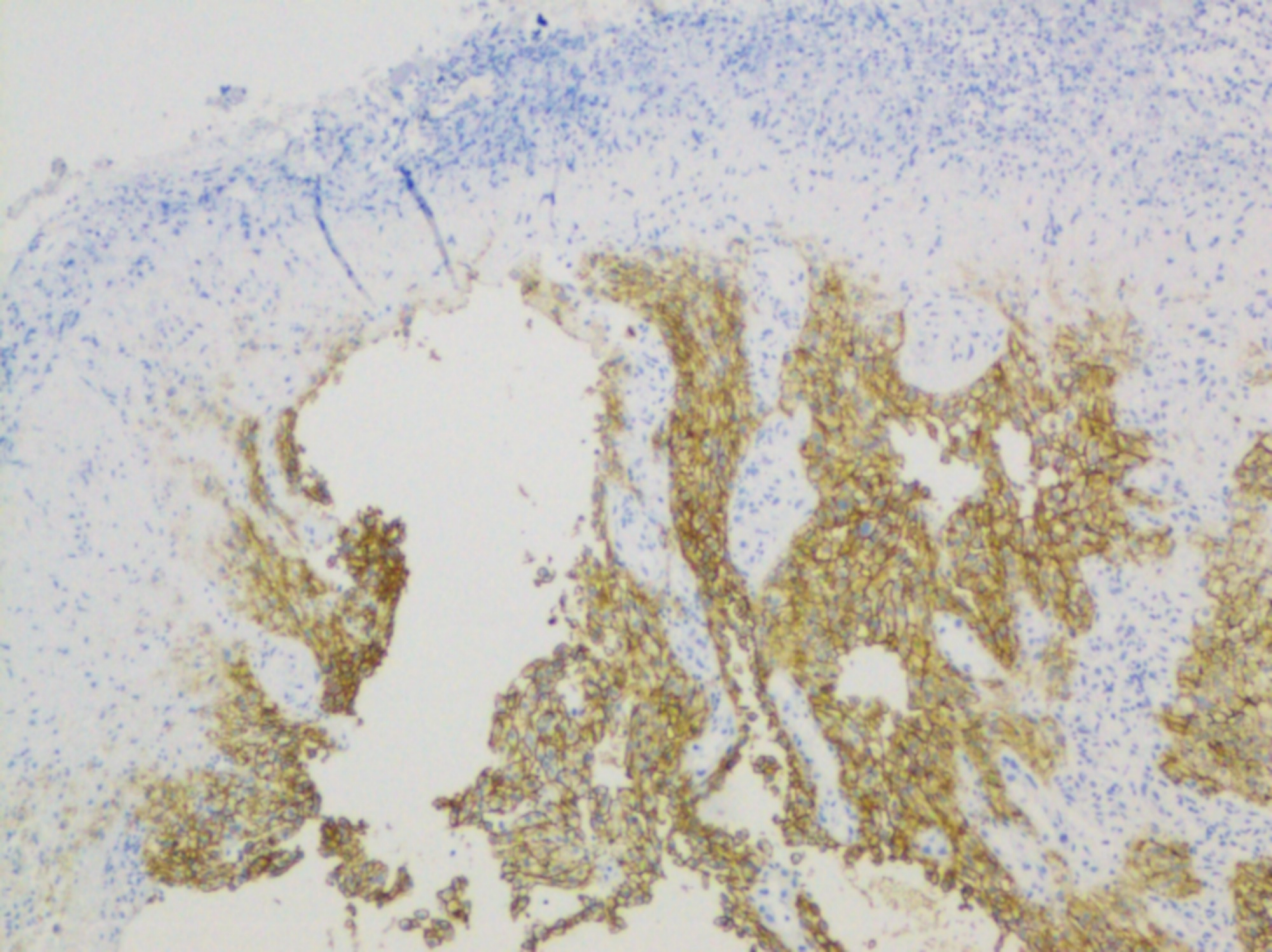
Histopathology from esophageal biopsy showing metastatic clear cell renal cell carcinoma using renal cell carcinoma (RCC) stain.

The histopathology report of esophageal mass revealed an erosive and ulcerated esophageal mucosa with underlying metastatic renal cell cancer of clear cell subtype (Figures 5-6).

Subsequently, pulmonary nodule was also confirmed as metastatic renal cell cancer. The patient was started on palliative radiotherapy and was discharged. On completion of radiotherapy two months later, his dysphagia had resolved. The patient is currently on chemotherapy with Sunitinib.

## Discussion

Metastasis to the esophagus is relatively uncommon. The underlying mechanism of metastasis can be a direct invasion of the adjacent organs (such as the larynx, hypopharynx, lungs, trachea/bronchus and stomach). It can also occur from distant organs like uterus or liver via hematogenous or lymphogenous routes [1]. Mizobuchi et al. reviewed the autopsy findings of 1,835 patients who died of cancer and reported metastatic esophageal cancer in 6.1% of patients. They found the site of the primary tumor as lung, breast, and stomach in 51, 14 and 13 cases respectively. In most patients with metastatic esophageal cancer, a direct invasion was noted [6].

Renal cell cancer has an abundant blood supply and metastasis is frequent and is detected in approximately 30% of renal cancer patients at first hospital visit. The relative ranking of site involved is lung > bone > regional lymph node > liver > Virchow’s node > brain [7]. Esophageal metastasis of renal cell cancer is very rare and so far only seven cases have been reported [8].

Though esophageal metastasis of RCC is very rare, however, it might become common in future as renal cancer is a slow growing tumor and improvement in therapeutic strategies will likely make survival longer. Recently occasional cases of metastasis of renal cancer to the pancreas were reported which used to be very rare [9]. It is highly likely that incidence of both pancreatic and esophageal metastasis of renal cancer will increase in future.

Diagnosis is confirmed by endoscopy, imaging, and histopathology. There are various treatment options which include surgical resection, chemotherapy and/or radiation. Surgical resection or endoscopic treatment is the first line of therapy and is indicated when the primary tumor is in the kidney and/or if there is a resectable solitary metastasis [1]. If the tumor is unresectable, multidisciplinary treatments including radiation and chemotherapy with molecular-targeting therapy agents like tyrosine-kinase inhibitors are indicated.

Prognostic factors proven to show better outcomes in patients with surgical resection of metastasis from RCC are short disease-free survival interval between treatment of primary tumor and development of metastasis, isolated metastasis, small size of metastasis to be resected, and the location of metastatic disease, as patients undergoing resection of lung metastasis have better outcomes than patients undergoing resection of brain metastasis [5, 6].

## Conclusions

Our case depicts the rare late involvement of esophagus from renal cell cancer. Such presentations should alert physicians to promptly assess atypical gastrointestinal symptoms in patients with prior history of renal cell cancer. We recommend further studies in a larger number of patients to develop more appropriate treatments for these patients.
